# Quality of life of cancer patients at palliative care units in developing countries: systematic review of the published literature

**DOI:** 10.1007/s11136-020-02633-z

**Published:** 2020-09-18

**Authors:** Dwi Gayatri, Ljupcho Efremov, Eva Johanna Kantelhardt, Rafael Mikolajczyk

**Affiliations:** 1 Institute for Medical Epidemiology, Biometrics and Informatics (IMEBI), Interdisciplinary Center for Health Sciences, Medical School of the Martin-Luther University Halle-Wittenberg, Magdeburger Strasse 8, 06097 Halle (Saale), Germany; 2 Department of Epidemiology, Faculty of Public Health, Universitas Indonesia, Depok, Indonesia; 3 Department of Gynecology, Medical School of the Martin-Luther University Halle-Wittenberg, Halle (Saale), Germany

**Keywords:** Systematic review, Quality of life, Palliative care, Advanced cancer, Developing country

## Abstract

**Purpose:**

This systematic review aims to summarize factors that influence the quality of life (QOL) of advanced cancer patients in palliative care (PC) in developing countries. Understanding this context in developing countries milieu is necessary; however, this outcome is rarely reported.

**Methods:**

Following the PRISMA guidelines, the electronic databases MEDLINE, Embase, CINAHL, and Web of Science were systematically searched using the search terms: QOL, cancer, PC, and names of all developing countries. Studies with less than ten subjects, qualitative or pilot studies, reviews, conference abstracts, and that reported validation of QOL questionnaires were excluded.

**Results:**

Fifty-five studies from 15 developing countries in the African (*n* = 5), Latin America and the Caribbean (*n* = 10), and Asian (*n* = 40) region were included in the narrative synthesis. 65.4% were cross-sectional, 27.3% were cohort studies, 7.3% were RCTs or quasi-experimental studies. Around 30 QOL factors were studied with 20 different types of QOL instruments. Advanced cancer patients who were older, married/ever married, participated in additional care within PC, used complementary and alternative medicine (CAM), and practiced spirituality/religiosity showed higher QOL score. Low educational level and high depression were associated with a lower QOL.

**Conclusion:**

Various factors affect QOL among cancer patients in PC. Patients valued the use of CAMs; however, the quality and safety aspects should be properly addressed. Important factors that influenced the QOL score were social and spiritual support. While there is a general need to develop PC strategies further, recognizing patients’ needs should be prioritized in national cancer programs.

**Electronic supplementary material:**

The online version of this article (10.1007/s11136-020-02633-z) contains supplementary material, which is available to authorized users.

## Introduction

Cancer remains a major public health problem in the world. In 2040, it is expected that 16.3 million people will live with cancer, most of them from low- and middle-income countries [1]. In these countries, the diagnosis for most cancers is frequently made at advanced stages when treatment options are limited or not accessible [2]. Cancer symptoms and treatment negatively affect patients’ quality of life (QOL) because of physical discomfort, mental stress, and economic pressure [3–6]. Therefore, in 1990 the World Health Organization (WHO) introduced the palliative care (PC) initiative, which represents medical care focused on improving the QOL of patients with a severe illness by treating symptoms through an interdisciplinary approach [7, 8]. PC improves QOL through prevention and relief of suffering by assessment, early identification, and treatment of pain, helping with physical or psychosocial problems, and providing spiritual support [8]. Cancer patients often continue treatments that no longer provide benefit to their health status, instead of aligning treatment strategies to improve their QOL. An effective PC strategy can provide appropriate support and symptom control for cancer patients [9].

PC and its accessibility remain limited in developing countries and certain considerations, such as differences between the needs of specific countries, cultural differences, different healthcare capacity and organization have to be taken into account [2]. Better understanding of the factors that improve cancer patients’ QOL in developing countries would be highly beneficial for initiating and/or strengthening PC implementation. However, most PC research originates from developed countries. Therefore, our systematic review aims to summarize evidence from the published literature on factors influencing cancer patients’ QOL in PC settings in developing countries.

## Methods

We followed a standard systematic review protocol, detailed in the Preferred Reporting Items for Systematic Reviews and Meta-Analyses (PRISMA) statement [10] and registered our systematic review with PROSPERO (CRD42019142567).

### Search strategy

We identified studies by searching MEDLINE, Embase, CINAHL, and Web of Science electronic databases. Search terms included ‘quality of life’, ‘cancer’, ‘palliative care’, and names of all developing countries. We followed the list of developing countries as published on the United Nations website (Online Resource 1) [11]. We used a broad search strategy to ensure a comprehensive review of the evidence and to capture all pertinent evidence. We supplemented our search strategy by manually reviewing references in the retrieved articles. We restricted our search to articles published in English between 1 January 1990 and 12 February 2019. The year 1990 was chosen, due to being the year of the WHO Palliative Care Initiative announcement [7].

### Eligibility criteria and study selection

Two reviewers performed the selection of studies. Studies were considered for an initial review if they met the following inclusion criteria: adult patients (≥ 18 years) with advanced cancer stage, in PC Units (PCUs), in developing countries, and assessing QOL/QOL domains as the outcome of interest. The advanced cancer stage was defined accordingly to the American Joint Committee on Cancer staging criteria [12]. PC is defined by the WHO as medical or non-medical methods meant not to cure, but to offer a support system for patients to live their life as actively as possible until death; any form of treatment that concentrates on reducing a patients’ symptoms or treatment-related side effects, improving QOL, and supporting patients and their families [13]. The primary outcomes were: (1) QOL score measured by QOL questionnaires [e.g. the European Organization of Research and Treatment for Cancer Quality of Life Questionnaire (EORTC QLQ), or the Functional Assessment of Cancer Therapy-General (FACT-G)]; (2) QOL domains e.g. functional scales (physical, role, emotional, cognitive, social functioning), symptoms scales (fatigue, nausea and vomiting, pain, dyspnea, insomnia, appetite loss, constipation, diarrhea, financial difficulties); or (3) symptoms/spirituality clusters, or specific symptoms (depression and anxiety).

The following exclusion criteria were used: studies with less than ten patients, qualitative or pilot studies, reviews, conference abstract, studies that included patients diagnosed with psychological disorders, and those that reported validation of QOL questionnaires. After removal of duplicates, titles and abstracts were screened by two authors independently (DG and LE), followed by assessment of the full text for selected studies to determine compliance with the inclusion criteria. Any disagreements were settled through discussion until a consensus was reached.

### Data extraction and quality assessment

The two reviewers independently extracted data from each study (year of publication, region, country, study design, population demographics, study sample size, cancer type, PCUs, reported factors linked to QOL/QOL domains, score of QOL/QOL domains, reported outcome of interest, and study quality assessment), and entered it in a standardized data extraction matrix. Factors that were positively or negatively associated with QOL/QOL domains are presented in a narrative synthesis. Outcomes including QOL score, as measured by the global health status of the EORTC-QLQ, overall well-being subscales, or overall mean QOL of the FACT-G were extracted. Data on other QOL domains, and symptoms/spirituality clusters, or specific symptoms were extracted when available. We performed critical appraisal using the quality assessment scale for cross-sectional studies [14], the Newcastle–Ottawa Quality Assessment Scale for cohort studies [15], and the risk of bias assessment tool by the Cochrane collaboration for randomized control trials (RCTs) or quasi-experimental studies [16] as described in more detail in Online Resource 2.

## Results

### Study selection

**Fig. 1:**
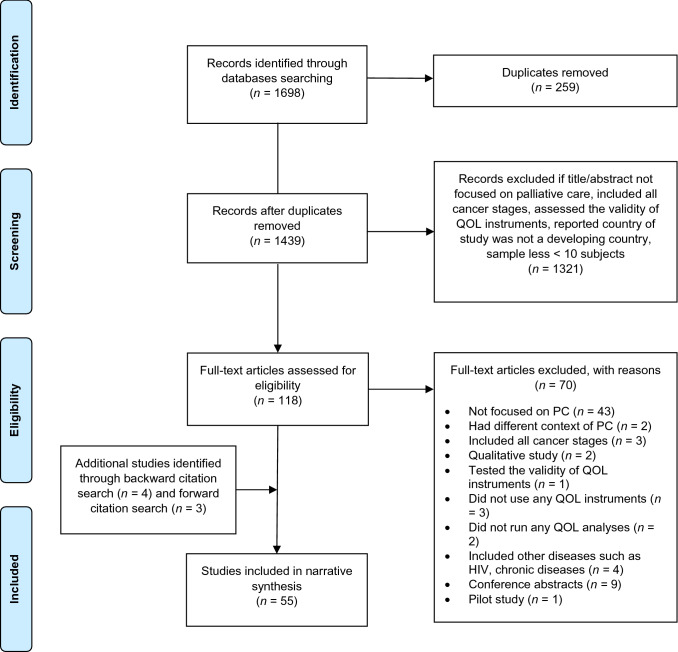
PRISMA flow diagram of study collection. *Source* Moher et al. [10]

The systematic search retrieved 1698 articles, after duplication removal 1439 articles (Fig. 1) were eligible for title and abstract screening using the predefined inclusion and exclusion criteria (Online Resource 1). We eliminated 1321 articles for not meeting the inclusion criteria. After screening the full text of 118 articles, 70 articles were excluded. Another seven articles were identified by searching reference lists of included articles. In total, 55 articles were included.

### Study characteristics and quality assessment

The number of advanced cancer patients analyzed in the selected studies ranged from 16 [17] to 1245 [18]. The most common study design was cross-sectional (36 studies), followed by 15 cohort studies, 2 RCTs, and 2 quasi-experimental studies. These studies were from 15 developing countries in the African region (Ethiopia, Kenya, and Malawi), Latin American and the Caribbean region (LAC) (Brazil), and Asian region (China, India, Indonesia, Jordan, Lebanon, Malaysia, Saudi Arabia, South Korea, Taiwan, Thailand, and Turkey). Geographically, most studies were from Asia (*n* = 40), followed by LAC (*n* = 10), and Africa (*n* = 5) (Table 1). Brazil, China, and India were countries with the highest number of studies (10, 9, and 9, respectively). While QOL research in developing countries were mostly conducted in hospital-based PC (*n* = 50), five studies did research on home-based PC (Online Resource 3). The results for cancer types and different QOL instruments are described in Online Resource 3. The quality assessment showed that from 36 cross-sectional studies, most studies (*n* = 32) had a low score (Table 1). Similarly, 2 of 4 RCTs and quasi-experimental studies had a low score. Only 5 of 15 cohort studies had a high score, as described in Online Resource 4.

**Table 1:** Characteristics of included studies

Author, year of publication	Country	Study design	Type of QOL questionnaire	Study sample size	Age	Sex	Type of cancer	PCUs	Reported outcome	Quality assessments
*Africa (n* = *5)*										
Bates et al., 2015 [78]	Malawi	Cross-sectional	N/A	72	Mean (min–max) 49.5 (20–80)	F: 72 (100%)	Cervical cancer 72 (100%)	Hospital-based	(cancer-related) Symptoms	Low
Kamau et al., 2007 [79]	Kenya	Cross-sectional	EORTC-QLQ-C30	152	20–29 = 7 (4.6%); 30–39 = 18 (11.8%); 40–49 = 45 (29.6%); 50–59 = 47 (30.9%); 60–69 = 26 (17.1%), ≥ 70 = 9 (5.9%)	F: 152 (100%)	Cervical cancer 152 (100%)	Hospital-based	Global health status	Low
Lakew et al., 2015 [54]	Ethiopia	Cross-sectional	Custom instrument	403	Mean (SD) 45.8 (11.3); < 35 = 41 (10.7%), ≥ 35 = 343 (89.3%)	N/A	All cancer types	Hospital-based	Physical well-being, social/family well-being	Low
Ogoncho et al., 2016 [55]	Kenya	Cross-sectional	MVQOLI	108	Mean (SD) 49.1 (4.1); min–max 18–71	F: 108 (100%)	Gynecological cancer: cervical 60 (56%), ovarian 26 (24%), endometrial 21 (19%)	Hospital-based	Overall QOL	Low
Ogoncho et al., 2015 [19]	Kenya	Cross-sectional	MVQOLI	108	Mean (min–max) 48 (18–72); 18–24 = 9 (8%); 35–55 = 39 (36%); 45–54 = 19 (18%); 55–64 = 22 (20%); > 65 = 19 (18%)	F: 108 (100%)	Gynecological cancer: cervical 60 (56%); ovarian 26 (24%); endometrial 21 (19%)	Hospital-based	Total QOL	Low
*Latin America and the Caribbean (n* = *10)*										
Alfano et al., 2014 [41]	Brazil	Cross-sectional	EORTC QLQ-C30; EORTC QLQ-BR23	126	Mean (SD) 51.4 (10.9)	F: 126 (100%)	Breast cancer 126 (100%)	Hospital-based	Global health status	Moderate
Avelino et al., 2015 [17]	Brazil	Cohort	EORTC QLQ C-30; QLQ-LC13	16	Median 63.7; < 65 = 6 (37.5%); ≥ 65 = 10 (62.5%)	F: 7 (43.8%); M: 9 (56.3%)	Non-small cell lung carcinoma 10 (62.5%)	Hospital-based	Global health status	Moderate
Camargos et al., 2015 [45]	Brazil	Cross-sectional	WHOQOL-Bref	525	< 30 = 13 (2.5%); 30–60 = 294 (56%); > 60 = 218 (41.5%)	F: 243 (46.3%); M: 282 (53.7%)	All cancer types 525 (100%)	Hospital-based	Overall QOL	Moderate
Carmo et al., 2017 [53]	Brazil	Intervention	EORTC QLQ-C15-PAL	63	Mean 52.9	F: 41 (65.1%); M: 22 (34.9%)	Breast cancer 18 (28.6%); cervix 9 (14.3%); colon and rectum 7 (11.1%); lung 7 (11.1%)	Hospital-based	Global health status	High
Mendes et al., 2014 [35]	Brazil	Cross-sectional	WHOQOL-BREF	56	Median (min–max) 65.5 (28–92)	F: 31 (55.4%); M: 25 (44.6%)	Gastrointestinal tract 20 (30.7%), respiratory tract 8 (14.3%), genitourinary tract 8 (14.3%), head and neck 6 (10.7%)	Hospital-based	QOL domains: physical, psychological, social relationships, environment	Low
Mendez et al., 2017 [36]	Brazil	Cohort	EORTC QLQ-C30, EORTC QLQ-BM22	35	Mean (min–max) 56 (22–80)	F: 23 (66%); M: 12 (34%)	Breast cancer 11 (31.4%), prostate 6 (17.1%)	Hospital-based	Global health status	Moderate
Paiva et al., 2012 [8]	Brazil	Cohort	ESAS	232	Median (min–max) 59 (18–88); mean (SD) 58.9 (12.8)	F: 112 (48%); M: 120 (52%)	Gastrointestinal 56 (24%); breast 41 (17%)	Hospital-based	Total symptom distress score (TSDS); symptoms score	High
Rigoni et al., 2016 [80]	Brazil	Cross-sectional	EORTC QLQ-C30; Coop/Wonca questionnaire	30	Mean 56.6	F: 2 (6.7%); M: 28 (93.3%)	Oropharynx 11 (36.7%); larynx 10 (33.3%)	Hospital-based	Global health status	Moderate
Rugno et al., 2014 [56]	Brazil	Cohort	EORTC QLQ-C30	87	Median (min–max) 56 (24–83); < 45 = 21 (24.1%); 45–65 = 50 (57.5%); > 65 = 16 (18.4%)	F: 87 (100%)	Breast 50 (50.7%); cervix 19 (21.8%); ovarian 14 (16.0%)	Hospital-based	Global health status	High
Rafael da Silva Ribeiro et al., 2015 [38]	Brazil	Cross-sectional	EORTC QLQ-C30	63	Median (min–max) 62 (33–84) patients in hospital-based PC vs 70 (49–90) in home-based PC	F: 38 (60.3%); M: 25 (39.7%)	Breast cancer 10 (15.9%), uterine cervix 6 (9.5%)	Hospital-based	Global health status	Low
*Asia (n* = *40)*										
Das et al., 2013 [29]	India	Cohort	FACT H and N	33	Mean (min–max) 57.8 (34–75)	F: 4 (12%); M: 29 (88%)	Head and neck cancer: oral cavity 11.9 (36%); hypopharynx and laryny 11.2 (34%)	Hospital-based	Physical, social, emotional, functional well-being	Moderate
Gandhi et al., 2014 [32]	India	Cross-sectional	EORTC QLQ-C15-PAL	100	Median (min–max) 55 (22–80)	F: 17 (17%); M: 83 (83%)	Head and neck cancer: oropharynx 46%; oral cavity except tongue 19%	Hospital-based	Global health status	Low
Ghoshal et al., 2016 [33]	India	Cohort	EORTC QLQ-C15-PAL	500	Median (SD) 52 (13.1); < 20 = 3 (0.6%); 21–40 = 101 (20.2%); 41–60 = 272 (54.4%); 61–80 = 118 (23.6%); > 80 = 6 (1.2%)	F: 204 (50.8%); M: 258 (51.6%)	Head and neck 116 (23.2%); gastro intestinal 106 (21.2%); gastro urinary 87 (17.4%)	Hospital-based	Global health status	High
Gielen et al., 2017 [27]	India	Cross-sectional	Hindi 36 spirituality items (custom made)	300	Mean (SD) 47.5 (12.4)	F: 148 (49.3%); M: 152 (50.7%)	Breast cancer 45 (15%), lung 26 (8.7%), gallbladder 15 (5%), rectum 13 (4.3%)	Hospital-based	Spiritual distress clusters	Low
Kandasamy et al., 2011 [47]	India	Cross-sectional	FACT-G, and FACT-pal	50	Mean (SD) 49.7 (10.2); min–max 17–64	F: 28 (56%); M: 22 (44%)	Oropharyngeal cancer 11 (22%), gynecological (cervix, endometrium, ovary) 11 (22%)	Hospital-based	QOL; subscales: physical well-being, social/family well-being, emotional well-being, functional well-being	Moderate
Mehta et al., 2008 [30]	India	Cohort	EORTC QLQ-C30	62	Mean (min–max) 62 (30–70)	F: 30 (48.4%); M: 32 (51.6%)	Carcinoma esophagus 62 (100%)	Hospital-based	Global health status	High
Prasad et al., 2015 [31]	India	Cohort	EORTC QLQ-C30	33	Median (min–max) 60 (35–78)	F: 12 (36.4%); M: 21 (63.6%)	Esophageal 33 (100%)	Hospital-based	Global health status	Moderate
Nayak et al., 2019 [37]	India	Cross-sectional	the QOL questionnaire version II (cancer institute quality of life questionnaire version II for cancer patients in India)	768	51–60 = 232 (30.2%); other age groups N/A	N/A	Head and neck cancer 308 (40.1%); breast/cervix/gastrointestinal tract/lung/colorectal were not reported in detailed	Hospital-based	General well-being score	Low
Palat et al., 2018 [81]	India	Cross-sectional	(unvalidated) POS, and HADS	76	Mean (SD) PC 48.6 (12.1) without contact to PC group vs 49.9 (16.2) with contact to PC group	F: 38 (50%); M: 38 (50%) both groups	Gastrointestinal 11 (14.4%); cervix 11 (14.4%); head and neck 10 (13.2%); lung 9 (13.2%)	Hospital-based	Pain intensity	Low
Aboshaiqah et al., 2016 [3]	Saudi Arabia	Cross-sectional	EORTC QLQ-C15-PAL	130	Mean (SD) 46.7 (16.5); min–max 17–86	F: 103 (79.2%); M: 27 (20.8%)	Breast cancer 69 (53.1%); colorectal 19 (14.6%); lung 15 (11.5%)	Hospital-based	Global health status	Moderate
Abu-Saad Huijer et al., 2012 [82]	Lebanon	Cross-sectional	EORTC QLQ-C30	200	Mean (SD) 54 (13.6); min–max 19–86; 13–34 = 17 (8.5%); 35–54 = 87 (43.5%); ≥ 55 = 96 (48%)	F: 126 (63%); M: 74 (37%)	Breast cancer 89 (44.5%); gastrointestinal system 36 (18%); blood 20 (10%)	Hospital-based	Symptoms prevalance	Low
Al-Zahrani et al., 2014 [83]	Saudi Arabia	Cross-sectional	AQSA	124	Mean (min–max) 56 (20–92)	F: 51 (41%); M: 73 (59%)	Breast cancer 34 (27.4%); head and neck 19 (15.3%); genitourinary 16 (12.9%)	Hospital-based	Pain score	Low
Bulbul et al., 2017 [18]	Turkey	Cross-sectional	ESAS	1245	Mean (SD) 61.8 (9.4)	F: 141 (11.3%); M: 1,104 (88.7%)	Lung cancer 1,245 (100%)	Hospital-based	Well-being	Moderate
Shamieh et al., 2017 [57]	Jordan	Cohort	ESAS	298	Mean (SD) 52.7(13.7)	F: 86 (47%); M: 96 (52%)	Breast cancer 40 (22%); lung 37 (20%); gastrointestinal 31 (17%)	Hospital-based	Symptoms	Moderate
Aamir et al., 2012 [28]	Malaysia	Cross-sectional	EORTC-QLQ-C30 and HADS	288	Mean (SD) 54 (15.7); min–max 20–85	F: 111 (38.5%); M: 177 (61.4%)	All cancer types	Hospital-based	Global health status	Low
Chaiviboontham, 2015 [42]	Thailand	Cross-sectional	SWBS	240	Mean (min–max) 56.1 (19–86)	F: 122 (50.8%); M: 118 (49.2%)	Gastrointestinal, breast, hepatobiliary, lung (no number reported)	Hospital-based	QOL	Moderate
Chan et al., 2012 [21]	China	Cross-sectional	MQOL-HK, HADS	53	Mean (SD) 62.11 (15.5); min–max 35–92	F: 53 (100%)	Gynecological cancer: ovary 29 (54.7%); cervix 13 (24.4%)	Hospital-based	Mean total QOL	Low
Chang et al., 2009 [25]	Taiwan	Cohort	SF-36	180	Mean (SD) 67.3 (13.1)	F: 57 (32%); M: 123 (68%)	Lung 39 (22%); colorectal 29 (16%); gastric 21 (12%)	Hospital-based	Survival time (as QOL proxy)	Moderate
Chui et al., 2009 [20]	China	Cross-sectional	MQOL	300	Mean (min–max) 67.5 (21–94)	F: 136 (45.3%); M: 163 (54.3%)	Lung, gastrointestinal are the most common out of 9 primary site of cancer (no number reported)	Hospital-based	Total QOL, QOL single item	Moderate
Cui et al., 2014 [26]	China	Cross-sectional	MQOL	531	18–44 = 48 (9%); 45–59 = 145 (27.3%); 60–74 = 164 (30.9%), > 75 = 174 (32.8%)	F: 234 (44.1%); M: 297 (55.9%)	All cancer types	Hospital-based	QOL	Moderate
Deng et al., 2015 [52]	China	Cohort	MQOL	630	Median (min–max) 62 (20–78)	F: 282 (44.8%); M: 348 (55.2%)	Lung 203 (32.2%); liver 67 (10.6%); gastric 52 (8.3%)	Hospital-based	Overall QOL	High
Ezat et al., 2014 [84]	Malaysia	Cross-sectional	SF-36 QOL	120	Mean (min–max) 57 (22–83)	F: 63 (52.5%); M: 57 (47.5%)	Lung 40 (33.4%); breast cancer 24 (20%); colon 19 (15.8%)	Hospital-based	QOL	Low
Fan et al., 2011 [48]	China	Cross-sectional	EORTC QLQ-C30	173	Mean (SD) 61.13 (12); min–max 19–86	F: 79 (45.7%); M: 94 (54.3%)	Lung 71 (41.0%); gastrointestinal 24 (13.9%); liver pancreas 24 (13.9%)	Home-based	Global health status	Moderate
Kim, 2014 [22]	South Korea	Cross-sectional	MQOL (the McMaster Quality of Life	52	Mean 49.2	F: 13 (25.5%); M: 38 (74.5%)	Lung 15 (28.9%), stomach, liver, gall bladder and leukemia each 5 (9.6%)	Hospital-based	Overall QOL	Moderate
Kim et al., 2013 [49]	South Korea	Cohort	EQ-VAS	262	Median (min–max) 60 (22–91)	F: 104 (39.7%); M: 158 (60.3%)	Colorectal 56 (21.4%); gastric 50 (19.1%); hepatobiliary 48 (18.3%)	Hospital-based	QOL	Moderate
Kristanti et al., 2017 [23]	Indonesia	Cohort	EORTC QLQ-C30	30	18–44 = 9 (30%); 45–54 = 10 (33%), > 55 = 11 (37%)	F: 22 (73%); M: 8 (27%)	Breast cancer 9 (30%); digestive (colon, recti, sigmoid) 5 (17%); gynecology (vulva, ovarian, cervical) 5 (17%)	Hospital-based	Global health status	High
Lau et al., 2013 [9]	China	Cross-sectional	QOLC-E	90	Mean (min–max) 64 (38–87)	F: 45 (50%); M: 45 (50%)	Respiratory 33 (36.7%); digestive-gastrointestinal 26 (28.9%); head and neck 10 (11.1%)	Hospital-based	Overall QOL; global QOL by single-item scale	Low
Lee et al., 2015 [34]	South Korea	Cohort	EORTC QLQ-C30	463	Mean (min–max) 57.3 (20–87)	F: 196 (42.3%); M: 267 (57.7%)	Stomach 83 (18%); colon 73 (15.8%); lung 67 (14.5%)	Hospital-based	Global health status	High
Lee et al., 2014 [85]	South Korea	Cohort	EORTC QLQ-C15-PAL	162	≥ 65 = 82 (50.6%); 40–65 = 75 (46.3%) < 40 5(3.1%)	F: 86 (53.1%); M: 76 (46.9%)	Lung 40 (24.7%); hepatobiliary 34 (21%); ovary/cervix of uterus 31 (19.1%)	Hospital-based	Physical functioning	Moderate
Lee et al., 2013 [50]	South Korea	Cohort	EORTC-QLQ-C30 and HADS	98	Mean (SD) 57.3 (10.9); 24–40 = 4 (4.1%); 41–60 = 50 (51.0%); 61–78 = 44 (44.9%)	F: 31 (31.6%); M: 67 (68.4%)	Stomach 44 (44.9%); lung 32 (32.6%)	Hospital-based	Overall QOL	Moderate
Li et al., 2014 [86]	China	Cross-sectional	EORTC QLQ-C30	109	Mean (SD) 69 (7)	F: 43 (39.4%); M: 66 (60.6%)	Esophageal and gastric 44 (40.3%); lung 19 (17.4%); liver 11 (10.1%)	Hospital-based	Role functioning	Moderate
Lua et al., 2011 [43]	Malaysia	Cross-sectional	MQOL	39	Mean (min–max) 55.9 (27–82)	F: 22 (56.4%); M: 17 (43.6%)	All cancer types	Hospital-based	Global health status	Low
Pokpalagon et al., 2012 [44]	Thailand	Cross-sectional	MVQOLI	180	Mean (min–max) 55.2 (20–84)	F: 109 (60.6%); M: 71 (39.4%)	Breast 51 (28.3%); hepatobiliary 32 (17.8%); lung 26 (14.4%); colorectal 19 (10.6%)	Hospital-based and home-based	Overall QOL	Moderate
Shahmoradi et al., 2012 [87]	Malaysia	Cross-sectional	HQLI	61	Mean (SD) 59.2 (12.5) min–max 18–74	F: 33 (54%); M: 28 (46%)	Breast 11 (18%); colon 8 (13.1%); rectum 8 (13.1%)	Home-based	Mean total score	Low
Tang et al., 2016 [51]	Taiwan	Cohort	MQOL	325	< 66 = 225 (69.2%); 66 + = 100 (30.8%)	F: 138 (42.5%); M: 187 (57.5%)	Stomach 61 (18.8%); liver 54 (16.6%); pancreas 49 (15.1%)	Hospital-based	QOL	High
Tsai et al., 2012 Taiwan [39]	Taiwan	Cohort	Custom made "symptom reporting form"	426	Median (min–max) 67 (27–93)	F: 212 (48.2%); M: 228 (51.8%)	Lung 89 (20.2%); liver 79 (18%); colon-rectum 47 (10.7%)	Hospital-based	Fatigue	Moderate
Wang et al., 2016 Taiwan [58]	Taiwan	Cross-sectional	FACT-G	85	Mean (SD) 59.5 (12.4)	F: 45 (52.9%); M: 40 (47.1%)	Gastrointestinal 24 (28.2%); head and neck, breast, and liver each 12 (14.1%)	Hospital-based	Total FACT-G score	Moderate
Wang et al., 2011 [40]	China	Cross-sectional	FACT-G	201	Mean (SD) 65.5 (12.8); min–max 30–89	F: 84 (41.8%); M: 117 (58.2%)	Colorectal 47 (23.4%); lung 40 (19.9%); esophageal and gastric 28 (13.9%)	Hospital-based	Total FACT-G score	Moderate
Yan, 2006 [24]	China	Cross-sectional	MQOL-HK	85	Mean (SD) 63.39 (13.2); min–max 39–93	F: 48 (56.5%); M: 37 (43.5%)	Lung 28 (32.9%); cervix/uterine/ovary 10 (11.7%)	Home-based	Total QOL score	Moderate
Yoon et al., 2018 [46]	South Korea	Cross-sectional	EORTC QLQ-C15-PAL	202	Mean 64.9	F: 99 (49.1%); M: 103 (50.9)	Lung 45 (22.3%); colon/rectal 41 (20.3%); liver/biliary tract 29 (14.4%)	Hospital-based	Overall QOL	Low

*QOL* quality of life, *PCUs* palliative care units, *N/A* not available, *F* female, *M* male, *SD* standard deviation, *EORTC-QLQ-C30* the European Organization for Research and Treatment of Cancer quality of life questionnaire of cancer patients, *EORTC QLQ-BR23* the European Organization for Research and Treatment of Cancer quality of life questionnaire of women with breast cancer, *EORTC QLQ-BM22* the European Organization for Research and Treatment of Cancer quality of life questionnaire of bone metastases, *EORTC QLQ-C15-PAL* the European Organization for Research and Treatment of Cancer quality of life questionnaire of palliative care, *MVQOLI* the Missoula Vitas Quality of Life Index, *WHOQOL-BREF* the World Health Organization quality of life instruments, *ESAS* the Edmonton Symptom Assessment, *FACT H and N* Functional Assessment of Cancer Therapy Head and Neck, *FACT-G* the functional assessment of cancer therapy-general, *FACT-pal* the functional assessment of cancer therapy-palliative care, *POS* the palliative care outcome scale, *HADS* the Hospital Anxiety and Depression Scale, *AQSA* the Arabic Questionnaire for Symptom Assessment, *SWBS* the spiritual well-being scale, *MQOL-HK* Mc Gill quality of life questionnaire Hong Kong Chinese version, *MQOL* the McMaster Quality of Life, *SF-36* Study Short-Form-36, *EQ-VAS* the EuroQoL-5 dimensions-visual analog scale, *QOLC-E* the quality of life concerns in the end of life questionnaire, *HQLI* the hospice quality of life index

### Factors associated with QOL in PCUs

Around 30 factors were reported in the 55 included studies (Table 2). These studies showed that factors assessed and linked to QOL/QOL domains in developing countries varied across the African, LAC, and Asian region (Tables 3, 4).

**Table 2:** Factors associated with quality of life in included studies

Author, year of publication	Factors studied	Better Outcome (QOL and/or QOL domains)	Poorer Outcome (QOL and/or QOL domains)	QOL and/or QOL domains Score
*Africa (n* = *5)*				
Bates et al., 2015 [78]	Palliative care interventions (pain medication)	Pain	N/A	N/A
Kamau et al., 2007 [79]	Patients’ perception of diagnosis and treatment on palliative radiotherapy	N/A	Overall QOL; overall physical health	N/A
Lakew et al., 2015 [54]	Use of PC services (counseling services, service brochure and benefit, books and videos library, relaxation class, drop counseling and support service, 24 h telephone support and cancer advisory, home nursing service, perform home activities, monitory allowances)	Physical well-being; social/family well-being	N/A	N/A
Ogoncho et al., 2016 [55]	Specific additional care within PCUs (pain relief service, management of other symptoms, psychological counseling, spiritual care)	Total QOL; interpersonal subscale	N/A	Mean (SD) total QOL = 17.2 (0.4); interpersonal subscale 5.3 (1.1)
Ogoncho et al., 2015 [19]	Age (> 65), occupational (formal employment status), monthly income (> 10,000 Kenyan shillings or equal to 99 US$), level of education (high), type of gynecological cancer, type of historic received cancer treatment	Total QOL	N/A	Positive improvement in mean (SD): age group 18–24 = 17 (1.2); 35–44 = 16 (3.3); 45–54 = 16 (4.1); 55–64 = 7 (4.7); > 65 = 21 (3.3); occupation: housewife = 6 (4.5); peasant farmer = 16 (4.7); casual worker = 16 (1.6); self-employed = 18 (3.6); formal employment = 20 (3.2)
*Latin America and the Caribbean (n* = *10)*				
Alfano et al., 2014 [41]	Body-mind interventions (mediation, yoga, acupuncture, relaxation, prayer, hypnotherapy, psychotherapy, and art therapy) as one of complementary and alternative medicine (CAM)‘s modalities	Sexual enjoyment; perspective for the future	Cognitive function	Median (P25-P75) sexual enjoyment = 66.7 (33.3–100); future perspective = 0.0 (0.0–66.7); cognitive function* n* = 66.7 (50.0–100)
	Biologically based practices (medicinal herbs, vitamins, minerals, food supplements, and probiotics) as one of CAM’s modalities	N/A	More frequent constipation	Median (P25-P75) constipation* n* = 0.0 (0.0–33.3)
Avelino et al., 2015 [17]	Chemotherapy cycles	Physical function; congnitive function; cancer-related symptoms e.g. pain, and appetite loss	N/A	Mean ± SD, median physical function* n* = 59.8 ± 27.7, 60.0 to 81.5 ± 20.9, 93.3; cognitive function* n* = 79.0 ± 35.9, 100.0 to 73.1 ± 30.1, 83.3; pai* n* = 60.4 ± 35.4, 58.4 to 78.2 ± 23.9, 83.3; appetite loss = 41.7 ± 46.4, 16.5 to 79.5 ± 39.8, 100.0
Camargos et al., 2015 [45]	Spirituality/religiosity (connection, meaning in life, admiration, wholeness and integration, spiritual strength, inner peace, hope and optimism, faith)	Global QOL; social domain; environmental domain	N/A	Mean (SD) global QOL = 78.0 (16.8); social domain* n* = 77.6 (17.2); environmental domain* n* = 75.8 (15.6)
Carmo et al., 2017 [53]	Psychosocial intervention based on the cognitive-behavioral therapy (CBT) techniques (psychoeducation on patient’s current clinical condition and the purpose of palliative care, the functioning of anxiety, technique to manage symptoms, and techniques for the detection and questioning of automatic thoughts as well as their influence and essential role in the triggering of emotions and behaviors)	Global health status	N/A	Mean (SD) global health status Arm A (received psychological intervention, received early PC intervention) = 76.3 (17.0); Arm B (received early PC intervention) = 72.7 (25.5); Arm C (standard cancer treatment) = 66.7 (29.5)
Mendes et al., 2014 [35]	Pain intensity	Environment domain	Physical domain	Scale value range from 0–100 environmental domain* n* = 70.7; physical domain* n* = 51.3
Mendez et al., 2017 [36]	Pain changes	Global health status	N/A	Median (IQR) global health status = 66 (50–100) to 33 (16–66)
Paiva et al., 2012 [8]	Anorexia, fatigue, nausea, pain, depression and anxiety, drowsiness, well-being, dyspnea	N/A	Total symptom distress score (TSDA)	Positive improvements in patients: anorexia = 90; fatigue = 112; nausea = 57; pai* n* = 133: positively improved from baseline and follow-up; depression* n* = 102; anxiety = 121; drowsiness = 98; well-being = 128; dyspnea = 56; TSDA = 232
Rigoni et al., 2016 [80]	Patients’ perception of pain, difficulty to detect problems, problem with social contact, use of analgesics, weight loss	N/A	Global health status	Mean ± SD, median global health status = 54.17 ± 24.93, 58.33
Rugno et al., 2014 [56]	Integrated care model (ICM)	Global health status; emotional functioning; social functioining; insomnia	N/A	Median score for global health status = 66.6; emotional functioning = 66.6; social functioning = 83.3; insomnia = 33.3
Rafael da Silva Ribeiro et al., 2015 [38]	Home-based palliative care	Global health status; functional scale; most of symptoms (except insomnia and diarrhea)	N/A	Global health status score (range from 0–100) = 57.1
*Asia (n = 40)*				
Das et al., 2013 [29]	Hypofractionated palliative radiotherapy (short duration)	Social well-being	N/A	Positive improvement: social well-being = 17.4 to 20.0
Gandhi et al., 2014 [32]	Symptoms, emotional functioning, physical functioning	Global health status	N/A	Mean global health status = 50.84
Ghoshal et al., 2016 [33]	Fatigue changes	Overall QOL; physical functioning; insomnia	N/A	Median (SD) Overall QOL = 50.0 (21.15) to 66.7 (26.58); physical function = 46.7 (24.08) to 60.0 (26.51); insomnia = 33.33 (30.98) to 0 (22.79)
Gielen et al., 2017 [27]	Sex (male), educational level (high), pain score (low)	(patients) Spirituality distress clusters	N/A	N/A
Kandasamy et al., 2011 [47]	Spiritual well-being	QOL; physical well-being; social family well-being; emotional well-being; family well-being; palliative well-being	N/A	N/A
Mehta et al., 2008 [30]	Combined palliative radiotherapy (intraluminal brachytherapy and external radiation)	Global health status	N/A	Mean QOL score Arm A (30 Gy/10 fractions/2 weeks XRT + 12 Gy ILBT (600 cGy per session × 2)) = 38 to 56; Arm B (30 Gy/10 fractions/2 weeks XRT) = 30 to 55; Arm C (20 Gy/5 fractions/1 week XRT) = 24 to 37
Prasad et al., 2015 [31]	Palliative radiotherapy	Global health status; dysphagia	N/A	Mean (SD), median global health status = 107.5 (10.1), 106 to 114.1 (7.5), 116; dysphagia = 4.1 (1.2), 4 to 2.4 (1.1), 3
Nayak et al., 2019 [37]	Symptoms, cognitive well-being (good), economic status (high)	Body image	QOL; physical and psychological domains	general well-being total score (mean ± SD) 32 (10.65 ± 3.23)
Palat et al., 2018 [81]	Contact with PC units	N/A	Pain	N/A
Aboshaiqah et al., 2016 [3]	Satisfaction care	Emotional function	N/A	N/A
Abu-Saad Huijer et al., 2012 [82]	Cognitive functioning	Global health status	N/A	Mean (SD) global health status = 58.46 (23.86)
Al-Zahrani et al., 2014 [83]	Age, sex, type of cancer, performance status, type of encounter in PC	Pain intensity	N/A	N/A
Bulbul et al., 2017 [18]	Age, body weight, weight loss, metastasis, symptom distress (pain, tiredness, drowsiness, lack of appetite, shortness of breath, depression, anxiety)	N/A	Well-being	N/A
Shamieh et al., 2017 [57]	Initial consultation provided by PC team	Symptoms: pain; fatigue; nausea; depression; anxiety; drowsiness; appetite; well-being; dyspnea; and sleep	N/A	Mean (SD) pain = 7 (1.8) to 6 (2.8); fatigue = 7 (1.8) to 6 (2.5); nausea = 7 (2.0) to 4 (3.7); depression = 7 (1.9) to 5 (3.2); anxiety = 7 (2.0) to 5 (3.0); drowsiness = 6 (1.7) to 5 (2.7); appetite = 7 (1.9) to 6 (3.0); well-being = 7 (1.8) to 6 (2.6); dyspnea = 6 (1.8) to 5 (3.2); sleep = 7 (1.8) to 5 (2.7)
Aamir et al., 2012 [28]	Depression, anxiety	N/A	Global health status	Mean ± SD global health status = 35.5 ± 10.9 (for depression); global health status = 34.3 ± 12.2 (for anxiety)
	Family function	N/A	Global health status	Mean ± SD global health status = 80.5 ± 14.7
Chaiviboontham, 2015 [42]	Spiritual well-being, a combination of PC strategy (pharmacological and psychosocial care, mind–body intervention, and spiritual care; physical management; and traditional medicine, herbs, and diet management)	QOL	N/A	N/A
Chan et al., 2012 [21]	Age (older)	QOL	N/A	Mean total score QOL = 0.683
	Depression, and anxiety	N/A	QOL	Mean total score QOL = -0.518 (for depression); Mean total score QOL = -0.278 (for anxiety)
Chang et al., 2009 [25]	Sex (female), KPS (high), overall symptoms severity (low)	N/A	Survival time (as a proxy of QOL)	N/A
Chui et al., 2009 [20]	Age (older), sex (female), marital status (had ever been married), physical functioning (higher)	QOL score	N/A	Mean total score QOL = 6.2 (range 0–10)
Cui et al., 2014 [26]	Sex (female), educational level (university +), number of children > 3), awareness of the disease (not aware at all), hospital size (tertiary)	MQOL total score	N/A	Mean ± SD QOL = 5.09 ± 0.90
Deng et al., 2015 [52]	Specific additional care within PCUs	QOL domains (physical, psychological, physical well-being, depressed, anxious, sad, fear of future, seeing life as a burden, existential well-being, personal existence, achieving life goals, life is worthwhile, self-content, closeness to people, world is caring, dignity, support, eating, sex); overall QOL	N/A	Mean (SD) physical = 6.21 (1.51) to 4.16 (2.06); psychological = 5.99 (2.05) to 4.89 (2.17); physical well-being = 7.03 (1.96) to 5.90 (2.41); depressed = 5.92 (2.54) to 4.83 (2.56); anxious = 6.24 (2.53) to 4.97 (2.58); sad = 5.90 (2.69) to 4.64 (2.61); fear of future = 4.93 (2.77) to 4.06 (2.61); seeing life as a burden = 5.94 (2.66) to 4.97 (2.69); existential well-being = 4.22 (1.89) to 3.59 (1.81); personal existence = 4.62 (2.52) to 4.10 (2.41); achieving life goals = 5.34 (2.69) to 4.78 (2.64); life is worthwhile = 4.53 (2.47) to 4.04 (2.39); self-content = 4.76 (2.71) to 4.17 (2.51); closeness to people = 2.92 (2.49) to 2.14 (2.13); world is caring = 2.97 (2.22) to 2.23 (2.04); dignity = 4.41 (2.98) to 3.68 (2.68); support = 6.55 (2.34) to 6.12 (2.53); eating = 6.34 (2.61) to 6.17 (2.84); sex = 6.37 (3.03) to 6.08 (3.12); overall QOL = 6.81 (2.27) to 5.87 (2.53)
Ezat et al., 2014 [84]	General satisfaction aspect, feeling at peace and having a sense of meaning in life	QOL	N/A	Mean (SD) = 63.96 (17.41)
	Time spent with doctor; accessibility	N/A	Physical component; mental component	Mean (SD) physical component = 42.24 (7.91); mental component = 44.93 (6.84)
Fan et al., 2011 [48]	Diagnosis awareness (aware)	Physical functioning and emotional functioning	N/A	Mean (SD) physical functioning = 38.45 (22.95); emotional functioning = 61.05 (19.27)
Kim, 2014 [22]	Age (> 70), sex (male), living with parents, not using analgesics, less symptoms other than pain	Overall QOL	N/A	N/A
Kim et al., 2013 [49]	Aware of terminal status (aware); primary cancer site (colorectal, gastric), lower depressive symptoms	QOL	N/A	Positive improvement in median (interquartile range) score: awareness of terminal status (unaware vs. aware) 60 (46.3–73.8) vs. 50 (30–70); primary cancer site colorectal 50 (40–70); gastric 60 (50–70); hepatobiliary 60 (46.3–60); pancreas 50 (30–70); head and neck 35 (30–70); metastatic to lung (no vs. yes) 60 (40–70) vs. 50 (35–70)
Kristanti et al., 2017 [23]	Basic skills training for family caregivers (educational package: instructional and informational video as well as demonstrations by nurse educators), age, sex (female), caregivers age and experience	Global health status; functional scales (emotional, and social function), symptoms/single items (fatigue, pain, dyspnea, insomnia, appetite loss, constipation, financial)	N/A	Mean (SD) global health status = 40.27 (17.79) to 56.94 (18.05); functional scales: emotional function = 63.33 (30.21) to 79.44 (26.77); social function = 20.56 (25.40) to 35.56 (33.82); symptoms: fatigue = 68.33 (24.20) to 56.29 (28.12); pain = 72.22 (33.99) to 57.22 (34.35); dyspnea = 38.89 (39.22) to 12.22 (28.34); insomnia = 57.78 (66.67) to 35.56 (36.04); appetite loss = 60.00 (39.53) to 44.44 (36.40); constipation = 32.22 (38.63) 20.00 (34.57); financial = 78.89 (29.66) to 65.55 (33.31)
Lau et al., 2013 [9]	Sex (female), walking ability (more ambulant)	Global QOL by single-item scale; overall QOL	N/A	Mean (SD) global QOL by single item = 5.72 (1.84); overall QOL = 6.27 (1.26)
Lee et al., 2015 [34]	Patient grouping (nonproblematic group)	Global health status; symptoms: fatigue; nausea; pain; dyspnea; insomnia; appetite loss; constipation; emotional function; cognitive function	N/A	Median survival days (CI95%) global health status = 101 (83–120); symptoms: fatigue = 93 (74–103); nausea = 77 (69–88); pain = 84 (69–98); dyspnea = 75 (69–88); insomnia = 77 (69–90); appetite loss = 94 (82–110); constipation = 77 (69–91); emotional function = 77 (69–93); cognitive function = 80 (69–93)
Lee et al., 2014 [85]	Performa status, cancer-related symptoms	Physical functioning	N/A	N/A
Lee et al., 2013 [50]	Awareness of incurable cancer status (aware)	Overall quality of life; role functioning; emotional functioning; social functioning	Depression; financial difficulty	N/A
Li et al., 2014 [86]	Role functioning; financial impact, fatigue; depression and anxiety	N/A	Global health status	Mean (SD) global health status = 39.82 (30.20)
Lua et al., 2011 [43]	Attitudes, beliefs and perceptions towards CIMT effectiveness in health maintenance, need for wider promotion, focus more on well-being, physical symptoms (CIMT user)	Global health status	N/A	Mean (SD), median global health status (for CIMT user) = 6.6 (2.8), 7.0; (for CIMT non-user) = 6.4 (1.9), 6.0
Pokpalagon et al., 2012 [44]	Having PC (non-pharmacological care strategy) at four institutions (religious organization/NGOs)	Overall QOL; well-being and transcendent	N/A	N/A
Shahmoradi et al., 2012 [87]	Functional status home-based PC	N/A	QOL	Mean ± SD QOL score = 189.9 ± 51.7
Tang et al., 2016 [51]	Prognostic awareness (accurate)	N/A	QOL; self-perceived sense of burden to others, anxiety	Mean (SD) QOL = 93.49 (25.58) to 75.05 (26.34)
Tsai et al., 2012 [39]	Symptoms (weakness, pain, anorexia, nausea/vomiting, dysphagia, restless/heat, abdominal fullness, constipation, dry mouth, dizziness), education of complexities in fatigue plus psychosocial and spiritual care	Fatigue	N/A	N/A
Wang et al., 2016 [58]	The meaning subscale (spiritual well-being), faith subscale	Total FACT-G score	N/A	Mean (SD) total score QOL = 78.15 (18.12)
Wang et al., 2011 [40]	Each 13 symptoms (fatigue, difficulty remembering, disturbed sleep, pain, poor appetite, distress, sadness, dry mouth, numbness, short of breath, drowsiness, nausea, vomiting), the sum score of 13 symptoms	N/A	Total FACT-G score	Mean (SD) total score QOL = 62.2 ± 16.8
	Psychological symptoms (distress, sadness)	Total FACT-G score	N/A	Mean (SD) total score QOL (for distress) = 2.84 (2.59); (for sadness) = 2.61 (2,56)
Yan, 2006 [24]	Age, palliative home care patients, using traditional Chinese Medicine (TCM)	Total QOL score; sexual functioning score, psychological score; existential score	N/A	Mean (SD) total QOL score = 6.61 (0.85); sexual function = 7.23 (2.78); psychological function = 7.14 (1.18); existential function = 6.46 (1.49)
	Pain intensity	N/A	Overall QOL; physical score, sexual functioning score	N/A
Yoon et al., 2018 [46]	Spiritual well-being	Overall QOL	N/A	N/A

Only reported results with *P* < 0.05 are included

*QOL* quality of life, *N/A* not available, *PC* palliative care, *SD* standard deviation, *CIMT* complementary indigenous Malay therapies, *NGOs* non-governmental organizations, *IQR* interquartile range, *Gy* Gray (unit of inonizing radiation dose in the International System Units), *XRT* external radiotherapy, *ILB* intraluminal brachytherapy, *KPS* the Karnofsky performance status

**Table 3:**
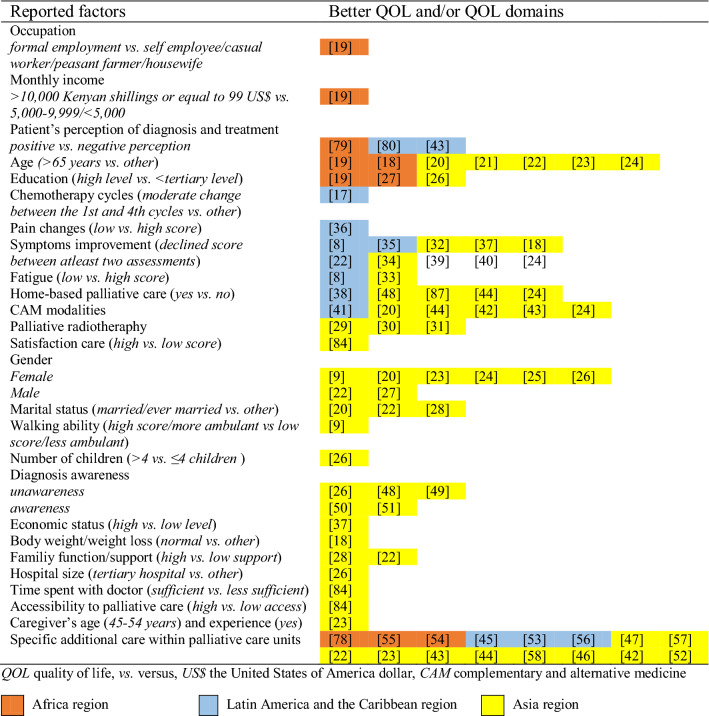
Factors associated with better quality of life in included studies by region

**Table 4:**
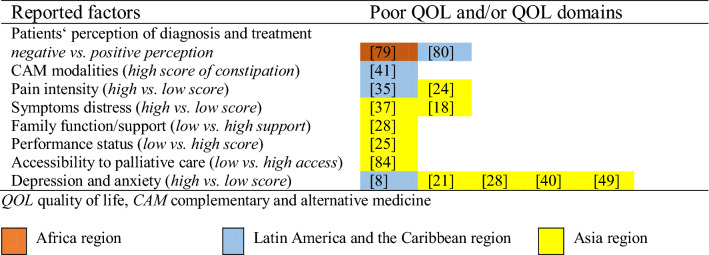
Factors associated with poor quality of life in included studies by region

#### Sociodemographic factors

The patients age ranged from 18 [19] to 94 years [20]. Studies from Africa [19] and Asia [18, 20–24] showed that older patients (> 65 years) had better QOL/QOL domains (psychological, existential, and support) compared to other age groups, which was related to positive coping mechanisms and social support from family and friends [20–22]. The reported gender proportions varied among the studies. While six studies showed that the proportion of male patients was higher than of female patients [9, 20, 22, 25–27], two studies demonstrated the opposite [23, 24]. Six Asian studies [9, 20, 23–26] reported that female cancer patients had better QOL/QOL domains (constipation or dyspnea in symptom function, physical functioning, sexual functioning, support, or spirituality clusters) than male patients, while one study in India [27] and one in South Korea [22] found the opposite results. Gielen et al. stated that in Indian tradition, women acted as the central providers of care in the families [27]. Having a life-threatening illness often results in the loss of the care role in the family, and contributes to a more distressing situation for Indian women [27]. In contrast, Taiwanese culture considered men as breadwinners and decision-makers in the family [25]. Therefore, family members often try to prolong a male patient’s life by sending them to the hospital for additional treatments, despite their terminal condition. Female cancer patients in Taiwan tended to receive PC at home and experienced a better QOL compared to male cancer patients [25]. Personality differences between male and female cancer patients were considered a key factor for the observed discrepancy [24]. Women were more expressive in their needs, more willing to seek and receive help from others compared to men. Consequently, they often received more support, which contributed to a better QOL [24].

Our review indicated that sociodemographic factors e.g. marital status (married/ever been married), number of children (> 4 children) [26], education (high level) [19, 26, 27], occupation (formal employment) [19], and income (high) [19] were linked to better scores in QOL/QOL domains. Evidence showed that patients who lived with family/spouse/children/parents were more likely to have better QOL/low score of depression and anxiety compared to those who lived alone [20, 22, 28]. Patients, who lived with a spouse, often received psychological and financial supports during their illness, which positively influenced their QOL [20, 22]. Moreover, patients with unsupported family members had a high score on anxiety and depression and subsequently poor QOL [28]. Only one study that assessed the association between the number of children and QOL reported that patients having > 4 children tended to have better QOL [26]. Children were considered as one of the key caregivers in developing countries’ culture, since taking care of sick parents is seen as a responsibility and not a burden [26]. A low proportion of cancer patients had a high level of education (range from 10 to 15.7%) [19, 27]. Low educational level was associated with decreased disease awareness, late screening, and late-stage diagnosis which leads to poor prognosis and low score of QOL [19]. Moreover, Gielen et al. reported that less-educated cancer patients often had low socioeconomic status (SES) in society [27]. As a consequence, they were more likely to have limited understanding of their disease, to show symptoms of depression, and have poor QOL [27]. Better education of patients is often linked to better job opportunities. For example, 17% of cancer patients who had formal employment tended to have higher social, psychological, spiritual, and QOL scores compared to those who were farmers and casual worker [19]. While formal employment was associated with adequate social support based on high-income earnings, low income was linked to poverty, low SES, and limited access to health care [19].

#### Important factors in clinical setting

Patients who underwent medical treatment e.g. chemotherapy cycles [17], palliative radiotherapy [29–31], and symptoms management therapy particularly for pain and fatigue [8, 18, 22, 24, 32–40], had positive association between these factors and QOL/QOL domains. For example, an Indian cohort study reported that short-course palliative radiotherapy schedule for inoperable head and neck cancer patients, which tended to improve social well-being, was seen favorably compared to the single conventional course of radiation [29]. Moreover, a Brazilian study, which assessed QOL change in four chemotherapy cycles showed that QOL/physical functioning was improved in advanced lung cancer patients [17]. Furthermore, Avelino et al. stated that chemotherapy at baseline assessment might improve QOL (small changes), physical and cognitive functioning [17]. Similarly, Mehta et al. emphasized that despite limited sample size which made the comparison between schedules underpowered, their study indicated that a combination of external beam radiotherapy with intraluminal brachytherapy in advanced esophageal cancer resulted in prolonged symptom palliation and a better QOL compared with the external radiotherapy alone [30].

#### Complementary and alternative medicine (CAM)

Six studies assessed the use of CAM to treat cancer patients in PC in Asia and LAC region [20, 24, 41–44]. A study reported 16% of cancer patients received Chinese medicine, and 14% claimed to take alternative therapy in addition to the standard cancer treatment, but no association with QOL was found [20]. Two studies found the use of traditional medicines such as complementary indigenous Malay therapies [43], and Chinese medicine showed a better QOL/existential subscale, or physical symptoms score [24]. Chaiviboontham [42] stated that nearly 63% of cancer patients tended to use a combination of pharmacological and non-pharmacological PC strategies e.g. psychosocial care, mind–body intervention, and spiritual care; physical management; and traditional medicine, herbs, and diet management to treat their illness. This was associated with the effectiveness of PC, with improvements in symptoms relief and spiritual well-being [42]. Similarly, Pokpalagon et al. [44] reported non-pharmacological PC strategies based on the use of herbal medicines showed a better overall QOL and well-being compared to only standard medical therapy. In contrast, Alfano et al. [41] found few CAM modalities e.g. body-mind intervention and food supplements that negatively affected QOL domains. For example, cancer patients who used body-mind interventions demonstrated poorer cognitive function compared to non-users. However, the same patients also reported greater sexual enjoyment, and positive perception for the future [41].

#### Spirituality/religiosity

Despite limited number of studies assessing spirituality/religiosity, various religions were reported, e.g. Buddhism, Protestantism, Catholicism, Evangelic, and Hinduism. Two studies reported that some patients had no religious affiliation [45, 46]. Four Asian studies [42, 44, 46, 47] and one Brazilian study [45] stated that spirituality/religiosity was associated with better scores for QOL/QOL domains. For example, the Brazilian study indicated that around 95% of patients believed spirituality/religiosity helps them during stressful situations, supports them during cancer treatment, is a useful coping mechanism, and is an important aspect for assessment by health professionals [45]. One Thai study showed higher QOL in cancer patients who were exposed to non-pharmacological care strategies (social supports, mediation, or reading Dharma book/bible and making merit) in PC organized by religious institution compared to those who were treated in community/university hospitals. Reading Dharma book/bible and making merit as part of Buddhism practice was the most common non-pharmacological strategy used, because this tended to bring happiness, peaceful life, and strengthening of the ability to face obstacles/misfortunes. It provided with a better stress coping mechanism and a better QOL [44]. Similarly, Kandasamy et al. stated that spirituality/religiosity has been closely linked to PC in India and was an important part of Indian cancer patients’ daily life, which acted as a coping stress mechanism, and could positively influence physical and psychological symptoms of distress [47]. A Korean study suggested that religion often provided comfort, a reason for living, a purpose in life, and harmony to cancer patients during their cancer treatments. Individual spiritual activities such as prayer, meditations, reading religious scriptures were beneficial for patients’ QOL/spiritual well-being, and in close relation with better hope and positive mood [46].

#### Diagnosis awareness

Diagnosis awareness was assessed by five Asian studies [26, 48–51]. In general, the proportion of patients’ awareness of their diagnosis was low, with a range from 17.5 to 50% [26, 48, 50], with only two studies showing a higher proportion [49, 51]. There are inconsistent reports if diagnosis awareness is associated with better QOL/QOL domains. For example, a South Korean study found a positive association between diagnosis awareness and QOL, role, emotional, and social functioning [50, 51]. Despite the positive association, Lee et al. emphasized that their result should be interpreted carefully [50]. In contrast, three studies reported opposite results [26, 48, 49]. Patients who were unaware of their diagnosis were more likely to have better physical and emotional functioning [48], and better overall QOL [26, 49] compared to those who knew their diagnosis. Fan et al. reported that the information non-disclosure gave a more hopeful outlook for patients, and increased the fighting spirit against the disease [48]. Cultural aspects were likely to play an important role for this non-disclosure. In some Asian cultures, a cancer diagnosis is a taboo concept, and patients often feel stigmatized and ashamed by their health condition; therefore, diagnosis unawareness could attribute to better physical and emotional functioning [48, 49].

#### Depression and anxiety

Five included studies showed that a high score for depression/anxiety is associated with poor QOL, physical well-being, emotional well-being, and functional well-being [8, 21, 28, 40, 49]. In our review, the proportion of advanced cancer patients feeling depressed and anxious ranged from 21.1 to 62%. This condition might decrease one’s hope and peace, lead to increase of physical pain, risk of suicide, and poor QOL [21, 28]. Chan et al. stated that other psychological domains of QOL e.g. being afraid of the future, feeling sad, and feeling a burden to others might intertwine with depression and anxiety [21]. Similarly, Kim et al. emphasized that depression is strongly associated with hopelessness, which negatively influences physical and psychospiritual well-being, and the immune system [49].

#### Common factors across regions

Some factors were found only in one specific region, while some commonly appeared within two or even in all three regions (Tables 3, 4). For example, included studies from the African region mostly explored sociodemographic factors e.g. occupation, income, age, and education (Online Resource 5), whereas the LAC’s studies provided information on factors in clinical settings and only one sociodemographic factor (patient’s perception of diagnosis and treatment). The included studies from the Asian region contributed to various factors in both clinical setting and sociodemographic aspects. The only common factor shared by all regions was specific additional care within PCUs e.g. symptoms management on pain and fatigue, spirituality/religiosity, psychosocial counseling, basic skills training for family caregivers, or exposure to integrated care management [22, 23, 42–47, 52–58].

## Discussion

This review indicates that in developing countries, cancer patients in PC who were older (> 65 years), married/ever married, had high educational level, used CAM, and practiced spiritual/religious activities were more likely to have higher scores in QOL/QOL domains. However, for patients with other characteristics e.g. younger patients, PCUs should be able to recognize and provide services that meet their needs [59]. Our review provides a broad perspective in terms of cancer types, geographical area, and factors that influence PC patients’ QOL. One previously published review focused on similar QOL context, but was limited only to the Asian region, non-PC, and female breast cancer survivors [60]. Our findings are in line with this study that individual and cultural perspectives, such as the use of CAMs, and spiritual/religious practices were key factors for a better QOL in cancer patients.

Advanced cancer patients experience a range of symptoms for which standard medical treatments may not provide sufficient relief [61]. Consequently, patients seek and use CAM as addition to standard cancer care. Our review showed that CAM modalities positively influence cancer patients’ QOL/QOL domains in PC. There are several possible explanations for this finding. First, in most developing countries standard cancer treatment options are limited, while CAM is available, accessible and affordable. One African study stated that most of the population in Africa lives in rural areas where standard healthcare services are limited [62]. This results in CAM being their primary source of healthcare. Second, the influence of cultural and historical factors is very important. Despite the well-established healthcare services in Singapore and South Korea, around 80% of their patients reported using CAM [63]. Moreover, most developing nations have their own traditional forms of healing stemming from their culture and history [64]. Last, as indicated by a British study, because of the failure of standard medical treatment or experiencing adverse effect from previous medical cancer treatment, patients are choosing CAM also in developed countries [65]. As demand for CAM increases worldwide [66], the safety and quality aspects remain an unaddressed issue [64]. CAM are considered as natural products and thus very safe, which is not necessarily true. Some CAMs might have a negative effect on patients and reduce the effectiveness of anticancer treatment [64]. Therefore, the WHO encourages CAM to be integrated and regulated by health service systems, particularly in PC, and evaluated with similar methods as standard treatment, such as clinical trials, to increase their quality and safety.

Having terminal illness is a highly depressing and anxiety-inducing condition. Our findings suggest that spirituality/religiosity positively affect cancer patients’ ability to cope with this situation. This can be explained by several mechanisms e.g. encouraging healthy behaviors, giving social supports, providing a belief system, offering coping mechanism, and influencing neuroendocrine and neuroimmunology pathways [64, 67]. Spirituality/religiosity also provides social support, facilitating a faster adaptation to the stressors [67]. A previous review of nearly 300 studies worldwide assessing the association between spirituality/religiosity and anxiety reported that around 50% of studies on this topic showed an inverse correlation [67]. A meta-analysis found that spirituality/religiosity-based interventions in developed countries had a positive effect on anxiety, stress levels, decreased alcohol use and late onset of depression [68]. According to one American longitudinal study, spirituality/religiosity is considered cost-effective [69], and helps give meaning to patients’ suffering and assists them in finding hope [70]. Therefore, recognizing spirituality/religiosity needs of cancer patients in PCUs by healthcare professionals is necessary.

Several individual characteristics such as age, gender, SES, and education are known to be linked to QOL domains as reported by previous studies from the USA [71], Turkey [72], and Asian countries [60]. However, there were some inconsistent findings, for example regarding diagnosis awareness. This inconsistency may be due to cultural differences across the regions. In many countries, disclosure of diagnosis and prognosis information of cancer patients is prohibited by the family or caregivers. This situation mostly happens because caregivers and/or health professionals assume that the disclosure of information on near death is detrimental to patients’ psychological wellbeing. However, patients’ acceptance following their diagnosis might positively influence their QOL. For example, traditional cultural values put a strong emphasis on concepts such as Buddhist and Confucian beliefs of enduring suffering [73]. Culture and ethnicity influences patients’ perspectives and experiences toward health and illness; therefore, assessing QOL domains especially related to acceptance of disease status is highly recommended.

There are several similarities, but also differences in factors affecting QOL of cancer patients between developed and developing countries [7]. One main difference is that the evidence reported by studies from developed countries is considered more robust due to a better methodological approach. While most studies in developed countries are commonly conducted as RCTs [74, 75], the cross-sectional study design is often used in developing countries. Factors with a positive effect on QOL are similar between developed and developing countries, such as use of CAM and spirituality/religiosity, in addition to the standard cancer treatment. However, direct comparison of these factors between countries remains a major issue; therefore, to achieve standardization of various non-medical cancer treatments further research in this context is needed.

Establishing PC services and incorporating them in national cancer programs might be challenging for most developing countries. PC development requires four important pillars: policy, education, medication availability, and implementation [76]. However, weaknesses in the healthcare system of developing countries limits PC implementation. Therefore, the WHO is strongly advocating for locally adapted PC services in all nations and emphasizing that access to these services is an important part of universal healthcare coverage schemes [59]. This idea is supported by evidence that PC is cost-effective and can decrease inefficient spending in healthcare for inappropriate hospital admissions, long hospital stay, inappropriate and ineffective use of medicine and/or treatment [59, 77]. Despite some challenges, our review puts emphasis on the possibility of improving QOL of advanced cancer patients even in limited-resource settings.

### Limitations

Over half of the articles had a low score in the quality assessment; therefore, the results should be carefully interpreted. The different types of QOL questionnaire in the included studies limit the comparability between studies. The included studies had various patient selection criteria, which might contribute to the inconsistency of some findings. Most studies had small sample size (< 300), and convenience samples, which makes the generalization of the results difficult.

## Conclusion

In developing countries, cancer patient’s sociodemographic characteristics (age, gender, marital status, and education) and cultural perspectives (the use of CAM, spirituality/religiosity) were key factors influencing QOL/QOL domains scores in PC. While CAM strategies and spiritual/religious practices were used and valued by cancer patients, its quality and safety aspects should be addressed with a proper biological assessment. Therefore, each country should recognize patients’ needs with more PC research and implement locally adapted strategies. Our narrative review should be interpreted as a guideline for stakeholders which factors should be prioritized.

## Electronic supplementary material

Below is the link to the electronic supplementary material.

Supplementary file1 (DOCX 36 kb)

## Data Availability

The data and material of this systematic review are available to the public upon request.
