# Supplementary material for: Quality of life of cancer patients at palliative care units in developing countries: systematic review of the published literature
Source: Qual Life Res. 2020 Sep 18;30(2):315–43. doi: 10.1007/s11136-020-02633-z (PMC7886760; doi:10.1007/s11136-020-02633-z)
Supplement: Supplementary file 1 — Supplementary file1 (DOCX 36 kb) [file 11136_2020_2633_MOESM1_ESM.docx]

Title: **Quality of life of cancer patients at palliative care units in developing countries: systematic review of the published literature**

Journal name: **Quality of Life Research**

Author names:

**Dwi Gayatri^1,2^, Ljupcho Efremov^1^, Eva Johanna Kantelhardt*^1,3^**, **Rafael Mikolajczyk^1^**

^1^ Institute for Medical Epidemiology, Biometrics and Informatics (IMEBI), Interdisciplinary Center for Health Sciences, Medical School of the Martin-Luther University Halle-Wittenberg, Halle (Saale), Germany.

Address: Magdeburger Strasse 8, D-06097 Halle (Saale), Germany

^2^ Department of Epidemiology, Faculty of Public Health, Universitas Indonesia, Depok, Indonesia.

^3^ Department of Gynecology, Medical School of the Martin-Luther University Halle-Wittenberg, Halle (Saale), Germany.

* Corresponding author (E-Mail: [eva.kantelhardt@uk-halle.de](mailto:rafael.mikolajczyk@uk-halle.de))

**Online Resource 1** Search strategy from MEDLINE filters: publication data from 1990 to 12 February 2019; English; human; adult (18+)

| Step(s) | Search strategy |
| --- | --- |
| 1 | exp "quality of life"/ |
| 2 | neoplasms/ |
| 3 | exp palliative care/ |
| 4 | 2 or 3 |
| 5 | brunei/ or cambodia/ or indonesia/ or laos/ or malaysia/ or myanmar/ or philippines/ or singapore/ or thailand/ or timor-leste/ or vietnam/ or bangladesh/ or bhutan/ or india/ or afghanistan/ or bahrain/ or iran/ or iraq/ or israel/ or jordan/ or kuwait/ or lebanon/ or oman/ or qatar/ or saudi arabia/ or syria/ or turkey/ or united arab emirates/ or yemen/ or nepal/ or pakistan/ or sri lanka/ or china/ or "republic of korea"/ or mongolia/ or taiwan/ |
| 6 | algeria/ or egypt/ or libya/ or morocco/ or tunisia/ or cameroon/ or central african republic/ or chad/ or congo/ or "democratic republic of the congo"/ or equatorial guinea/ or gabon/ or "sao tome and principe"/ or burundi/ or djibouti/ or eritrea/ or ethiopia/ or kenya/ or rwanda/ or somalia/ or sudan/ or tanzania/ or uganda/ or angola/ or botswana/ or lesotho/ or malawi/ or mozambique/ or namibia/ or south africa/ or swaziland/ or zambia/ or zimbabwe/ or benin/ or burkina faso/ or cabo verde/ or cote d'ivoire/ or gambia/ or ghana/ or guinea/ or guinea-bissau/ or liberia/ or mali/ or mauritania/ or niger/ or nigeria/ or senegal/ or sierra leone/ or togo/ |
| 7 | bahamas/ or barbados/ or cuba/ or dominican republic/ or haiti/ or jamaica/ or "trinidad and tobago"/ or belize/ or costa rica/ or el salvador/ or guatemala/ or honduras/ or nicaragua/ or panama/ or mexico/ or argentina/ or bolivia/ or brazil/ or chile/ or colombia/ or ecuador/ or guyana/ or paraguay/ or peru/ or suriname/ or uruguay/ or venezuela/ |
| 8 | 5 or 6 or 7 |
| 9 | 1 and 4 and 8 |

**Online Resource 2** Quality assessment in the methods and results section

**Methods**

Critical appraisal was performed using the quality assessment scale for cross-sectional studies [14], the Newcastle-Ottawa Quality Assessment Scale for cohort studies [15], and the risk of bias assessment tool by the Cochrane collaboration for randomized control trials (RCTs) or quasi-experimental studies [16]. We assessed the quality of each cross-sectional study based on three criteria (Online Resource 6). Two items on sample selection, one on comparability, and two on outcome measurement were used to assess cohorts’ study quality (Online Resource 4). The maximum score was 8 for each cross-sectional study, and 6 for each cohort study. Each cross-sectional study was rated low when it scored between 0-3, moderate for having a score of 4-6, and high with a score 7-8. For the cohort study, each was determined as low, moderate, and high quality when scoring 0-2, 3-4, and 5-6, respectively. RCTs and quasi-experimental studies were assessed through the standard criteria of risk of bias [16]. We checked each criterion in the studies and assessed them as low, unclear, or high risk of bias (Online Resource 7).

**Results**

Of 36 studies with a cross-sectional design, none received the maximum score of the quality assessment (Table 1). Most studies (*n* = 32) showed low score owing to convenience sampling, and small sample size (<300). Most studies (*n* = 29) described their study subjects which resulted in a high score on this aspect. In total, 8 studies failed to report confidence intervals or standard errors, and 16 had low score in the outcome category (adequate response rate) (Online Resource 6). Five of 15 cohort studies had good quality with a score of 5-6. Most studies (*n* = 12) were unable to have a representative sample of their target population (selection category). Five had inadequate response rate (outcome category), and did not describe the lost to follow up (Online Resource 4). Of 4 RCTs and quasi-experimental studies, 2 were at high risk for the blinding of participants and personnel criteria, lack of outcome assessment blinding and failed to conduct subject randomization as well as concealment (Online Resource 7) [23,52].

**Online Resource 3** Cancer types; Different instrument for QOL; Palliative care units in the results section

**Results**

*Cancer types*

Cancer types of included studies varied among regions (Table 1). For example, gynecological cancers (cervical, ovarian, and endometrial cancer) and breast cancer appeared in all three regions. QOL measurements for other types such as gastrointestinal, lung, head and neck, and colorectal cancer were studied infrequently. There are five studies (three in Asia, one in Africa and LAC) which enrolled patients with any cancer type in their study without providing specific information about the cancer type [26,28,43,45,54]. In the African region, only QOL in gynecological cancers was evaluated [19,55,78,79].

*Different instruments for QOL*

The number and type of QOL instruments varied, but the most common instrument in all regions was the EORTC QLQ-C30 (*n* = 22 studies) and its variants such as EORTC QLQ-C15-PAL (*n* = 6). The FACT-G questionnaire was used by four studies only in the Asia region [29,40,47,58]. The WHO QOL instruments (WHOQOL-BREF) were used by two Brazilian studies [35,45]. Only six studies (one in LAC region [53], five in Asia [3,32,33,46,85]) assessed QOL of advanced cancer patients in PC with the proper questionnaire such as the EORTC QLQ-C15-PAL, and one Indian study used FACT-pal [47]. We noted that few studies implemented custom-made instruments [39,54].

*Palliative care units (PCUs)*

Hospital-based PC (*n* = 50) in cancer hospitals, teaching hospitals, and national hospitals was reported as the most frequent setting (Tabel 1). Only five studies (Brazilian [35], Malaysian [87], Thai [44], and two Chinese studies [24,48]) conducted research on home-based PC. The percentage of patients who received home-based PC ranged from 24.4 to 84.7%. While the home-based PC in the Thai study was financially self-supported through donations [44], the other four studies offered home-based PC service affiliated either to a teaching university hospital in Brazil [38], a regional hospital in Hong Kong [24], or assisted by the home care service in China [48] and Malysia [87]. Only the study from Thailand explained more about its home-based PC approach, which was predominantly based on religion and herbal medicine. It implemented religious practices to increase patients’ faith, hope, and acceptance of life. A sense of sharing and healing was evident as volunteers, patients, and families participated together in activities.

**Online Resource 4** Quality assessment of 15 cohort studies

| **Author, year** | **Representativeness of the exsposed cohort^1^** | **Ascertainment of exposure^2^** | **Comparability of cohorts on the basis of the design or analysis^3^** | **Assessment of outcome^4^** | **Adequacy of follow up of cohorts^5^** | **Maximun score = 6** |
| --- | --- | --- | --- | --- | --- | --- |
| Avelino et al., 2015 | 0 | 1 | 1 | 1 | 1 | 4 |
| Chang et al., 2009 | 0 | 1 | 2 | 1 | 0 | 4 |
| Das et al., 2013 | 0 | 1 | 1 | 1 | 1 | 4 |
| Ghoshal et al., 2016 | 0 | 1 | 2 | 1 | 1 | 5 |
| Kim et al., 2013 | 0 | 1 | 1 | 1 | 1 | 4 |
| Lee et al., 2013 | 0 | 1 | 2 | 1 | 0 | 4 |
| Lee et al., 2014 | 0 | 1 | 1 | 1 | 1 | 4 |
| Lee et al., 2015 | 1 | 1 | 2 | 1 | 1 | 6 |
| Mendez et al., 2017 | 0 | 1 | 1 | 1 | 1 | 4 |
| Paiva et al., 2012 | 0 | 1 | 2 | 1 | 1 | 5 |
| Prasad et al., 2015 | 0 | 1 | 1 | 1 | 0 | 3 |
| Rugno et al., 2014 | 0 | 1 | 2 | 1 | 1 | 5 |
| Shamieh et al., 2017 | 0 | 1 | 1 | 1 | 0 | 3 |
| Tang et al., 2016 | 1 | 1 | 1 | 1 | 1 | 5 |
| Tsai et al., 2012 | 1 | 1 | 2 | 1 | 0 | 5 |

^1^ Selection criteria: a score of 1 is assigned if sample is truly or somewhat representative of the average advanced breast cancer patients in the community studied.

^2^ Selection criteria: a score of 1 is assigned if exposure information is from a secure record or structure interview where appropriate.

^3^ Comparability criteria: a score of 1 is assigned if baseline time is controlled for and an additional score of 1 is assigned if additional factor(s), mainly stage or age are accounted for.

^4^ Outcome criteria: a score of 1 is assigned if HRQL/QOL is assessed by self-reporter or by trained interviewer.

^5^ Outcome criteria: a score of 1 is assigned if all participants are accounted for or follow-up rate is ≥70% and description of lost to follow-up is described.

**Online Resource 5** Factors associated with quality of life of included studies based on regions in developing countries

| **Regions** | **Africa** | **Latin America and the Caribbean** | **Asia** | **All regions** |
| --- | --- | --- | --- | --- |
| **Africa** | - Occupation (formal employment status) [19] - Monthly income (>10,000 Kenyan Shillings or equal to 99 US$) [19] |  |  |  |
| **Latin America and the Caribbean** | - Patient’s perception of diagnosis and treatment [43,79,80] | - Chemotherapy cycles [17] - Pain changes [17,36] |  |  |
| **Asia** | - Age (older) [18-24] - Education (higher level) [19,26,27] | - Symptoms improvement [8,18,22,24,32,34,35,37,39,40] - Fatigue [8,33] - Home-based PC [24,38,44,48,87] - CAM* [20,24,41-44] - Depression and anxiety* [8,21,28,40,49] | - Hypofractionated palliative radiotherapy [29] - Combined palliative radiotherapy [30] - Palliative radiotherapy [31] - Satisfaction care [84] - Gender* [9,20,22-27] - Marital status [20] - Walking ability [9] - Number of children [26] - Diagnosis awareness* [26,48-51] - Economic status [37] - Body weight/weight loss [18] - Family function/living with parents [22,28] - Hospital size (tertiary) [26] - Time spent with doctor [84] - Accessibility [84] - Caregiver’s age, and experiences [23] |  |
| **All regions** |  |  |  | - Specific additional care within PCUs e.g. pain relief service, management of other symptoms, psychosocial counseling, spiritual care, PC service utilization, ICM, spirituality/religiosity/spiritual well-being, psychosocial intervention, pain management, initial consultation, basic skills training for family caregivers [22,23,42-47,52-58,78] |

*inconsistent findings of included studies within each or between regions

*PC* palliative care, *US$* the United States of America dollar, *CAM* complementary and alternative medicine, *PCUs* palliative care units, *ICM* integrated care model

**Online Resource 6** Quality assessment of 36 cross-sectional studies

| **Author, year** | **Random sample or whole population^1^** | **unbiased sampling frame^2^** | **adequate sample size (>300)^3^** | **Measures were standard^4^** | **Study subjects described^5^** | **Outcomes measured by unbiased assessors^6^** | **Adequate response rate (70%), refused described^7^** | **Confidence intervals, subgroup analysis^8^** | **Maximum score = 8** |
| --- | --- | --- | --- | --- | --- | --- | --- | --- | --- |
| Aamir et al., 2012 | 0 | 0 | 0 | 1 | 1 | 1 | 0 | 0 | 3 |
| Aboshaiqah et al., 2016 | 0 | 0 | 0 | 1 | 1 | 1 | 1 | 1 | 5 |
| Abu-Saad Huijer et al., 2012 | 0 | 0 | 0 | 1 | 1 | 1 | 1 | 0 | 4 |
| Alfano et al., 2014 | 0 | 0 | 0 | 1 | 1 | 1 | 1 | 1 | 5 |
| Al-Zahrani et al., 2014 | 0 | 0 | 0 | 1 | 1 | 1 | 0 | 0 | 3 |
| Bates et al., 2015 | 0 | 0 | 0 | 0 | 1 | 0 | 0 | 0 | 1 |
| Bulbul et al., 2017 | 0 | 0 | 1 | 1 | 1 | 1 | 1 | 1 | 6 |
| Camargos et al., 2015 | 0 | 0 | 1 | 1 | 1 | 1 | 1 | 1 | 6 |
| Chaiviboontham, 2015 | 0 | 0 | 0 | 1 | 1 | 1 | 1 | 1 | 5 |
| Chan et al., 2012 | 0 | 0 | 0 | 1 | 1 | 1 | 0 | 1 | 4 |
| Chui et al., 2009 | 0 | 0 | 0 | 1 | 1 | 1 | 1 | 1 | 5 |
| Cui et al., 2014 | 0 | 0 | 1 | 1 | 1 | 1 | 1 | 1 | 6 |
| Ezat et al., 2014 | 0 | 0 | 0 | 1 | 1 | 1 | 0 | 1 | 4 |
| Fan et al., 2011 | 0 | 0 | 0 | 1 | 1 | 1 | 1 | 1 | 5 |
| Gandhi et al., 2014 | 0 | 0 | 0 | 1 | 0 | 1 | 1 | 1 | 4 |
| Gielen et al., 2017 | 0 | 0 | 0 | 1 | 0 | 1 | 1 | 1 | 4 |
| Kamau et al., 2007 | 0 | 0 | 0 | 1 | 1 | 1 | 0 | 1 | 4 |
| Kandasamy et al., 2011 | 0 | 0 | 0 | 1 | 1 | 1 | 1 | 1 | 5 |
| Kim., 2014 | 0 | 0 | 0 | 1 | 1 | 1 | 1 | 1 | 5 |
| Lakew et al., 2015 | 0 | 0 | 0 | 0 | 0 | 1 | 1 | 1 | 3 |
| Lau et al., 2013 | 0 | 0 | 0 | 1 | 1 | 1 | 0 | 1 | 4 |
| Li et al., 2014 | 0 | 0 | 0 | 1 | 1 | 1 | 1 | 1 | 5 |
| Lua et al., 2011 | 0 | 0 | 0 | 1 | 1 | 1 | 0 | 0 | 3 |
| Mendes et al., 2014 | 0 | 0 | 0 | 1 | 0 | 1 | 1 | 1 | 4 |
| Nayak et al., 2019 | 0 | 0 | 1 | 1 | 0 | 0 | 0 | 0 | 2 |
| Ogoncho et al., 2015* | 0 | 0 | 0 | 1 | 0 | 0 | 0 | 1 | 2 |
| Ogoncho et al., 2016* | 0 | 0 | 0 | 1 | 1 | 1 | 0 | 1 | 4 |
| Palat et al., 2018 | 0 | 0 | 0 | 1 | 1 | 1 | 0 | 1 | 4 |
| Pokpalagon et al., 2012 | 0 | 0 | 0 | 1 | 1 | 1 | 1 | 1 | 5 |
| Rafael da Silva Ribeiro et al., 2015 | 0 | 0 | 0 | 1 | 0 | 1 | 0 | 0 | 2 |
| Rigoni et al., 2016 | 0 | 0 | 0 | 1 | 1 | 1 | 1 | 1 | 5 |
| Shahmoradi et al., 2012 | 0 | 0 | 0 | 1 | 1 | 1 | 0 | 0 | 3 |
| Wang et al., 2011 | 0 | 0 | 0 | 1 | 1 | 1 | 1 | 1 | 5 |
| Wang et al., 2016 | 0 | 0 | 0 | 1 | 1 | 1 | 1 | 1 | 5 |
| Yan, 2006 | 0 | 0 | 0 | 1 | 1 | 1 | 0 | 1 | 4 |
| Yoon et al., 2018 | 0 | 0 | 0 | 1 | 1 | 1 | 0 | 1 | 4 |

* Same data set and author, different outcome

^1^ Selection criteria: a score of 1 is assigned if sample is a random selection or the whole population of the community studied.

^2^ Selection criteria: a score of 1 is assigned if an unbiased sampling frame was used.

^3^ Selection criteria: a score of 1 is assigned if sample is >300.

^4^ Selection criteria: a score of 1 is assigned if exposure information is from a secure record or structured interview where appropriate.

^5^ Comparability criteria: a score of 1 is assigned if participants are described. However, studies that meet <50% of the criteria would be considered as having a low score

^6^ Outcome criteria: a score of 1 is assigned if HRQL/QOL is assessed by self-report or by trained interviewer. (Outcome)

^7^ Outcome criteria: a score of 1 is assigned if all participants are accounted for or follow-up rate is ≥70% and description of lost to follow-up is described.

^8^ Outcome criteria: a score of 1 is assigned if confidence intervals or standard errors are reported.

**Online Resource 7** Quality assessment of 2 RCTs and 2 quasi-experimental studies

| **Author, year** | **Random sequence generation (selection bias)^1^** | **Allocation concealment (selection bias)^2^** | **Blinding of participants and personnel (performance bias)^3^** | **Blinding of outcome assessment (detection bias) self-reported outcomes^4^** | **Blinding of outcome assessment (detection bias) Reaction time^5^** | **Incomplete outcome data (attrition bias)^6^** | **Selective reporting (reporting bias)^7^** | **Other bias^8^** | **Resume** |
| --- | --- | --- | --- | --- | --- | --- | --- | --- | --- |
| Deng et al., 2015 | High risk | High risk | High risk | High risk | High risk | Low risk | Unclear risk | Unclear risk | High risk |
| do Carmo et al., 2017 | Low risk | Low risk | Low risk | Low risk | Low risk | Low risk | Low risk | Unclear risk | Low risk |
| Kristanti et al., 2017 | High risk | High risk | High risk | High risk | High risk | Low risk | Unclear risk | Unclear risk | High risk |
| Mehta et al., 2008 | High risk | High risk | Low risk | Low risk | Low risk | Low risk | Low risk | Unclear risk | Low risk |

^1^ Selection bias (biased allocation to interventions) due to inadequate generation of a randomized sequence. Low risk is rated if a random component in the sequence generation process is described; high risk is rated if a non-random component in the sequecnce process is described; unclear risk is rated if insufficient information about the sequence generation process to permit judgement of low risk or high risk

^2^ Selection bias (biased allocation to interventions) due to inadequate concealment of allocation prior to assignment. Low risk is rated if both subjects and investigators could not foresee assignment because the use of proper methods to conceal allocation. High risk is rated if subjects of inverstigators could possibly foresee assignments and thus introduce selection bias. Unclear risk is rated if insufficient information to permit judgement of low risk or high risk

^3^ Performance bias due to knowledge of the allocated interventions by participants and personnel during the study. Low risk is rated if no blinding or incomplete blinding, but the review authors judge that the outcome is not likely to be influenced by lack of blinding; or blinding of participants and key study personnel ensured, and unlikely that the blinding could have been broken. High risk is rated if no blinding or incomplete blinding, and the outcome is likely to be influenced by lack of blinding; or blinding of key study participants and personnel attempted, but likely that the blinding could have been broken, and the outcome is likely to be influenced by lack of blinding. Unclear risk is rated if insufficient information to permit judgement of low risk or high risk; or the study did not address this outcome.

^4,5^ Detection bias due to knowledge of the allocated interventions by outcome assessors. Low risk is rated if no blinding of outcome assessment, but the review authors judge that the outcome measurement is not likely to be influenced by lack of blinding; or blinding of outcome assessment ensured, and unlikely that the blinding could have been broken. High risk is rated if no blinding of outcome assessment, and the outcome measurement is likely to be influenced by lack of blinding; or blinding of outcome assessment, but likely that the blinding could have been broken, and the outcome measurement is likely to be influenced by lack of blinding. Unclear risk is rated if insufficient information to permit judgement of low risk or high risk; or the study did not address this outcome.

^6^ Attrition bias due to amount, nature or handling of incomplete outcome data. Low risk is rated if no missing outcome data; reasons for missing outcome data unlikely to be related to true outcome (for survival data, censoring unlikely to be introducing bias); missing outcome data balanced in numbers across intervention groups, with similar reasons for missing data across groups; for dichotomous outcome data, the proportion of missing outcomes compared with observed event risk not enough to have a clinically relevant impact on the intervention effect estimate; for continuous outcome data, plausible effect size (difference in means or standardized difference in means) among missing outcomes not enough to have a clinically relevant impact on observed effect size; missing data have been imputed using appropriate methods. High risk is rated if reason for missing outcome data likely to be related to true outcome, with either imbalance in numbers or reasons for missing data across intervention groups; for dichotomous outcome data, the proportion of missing outcomes compared with observed event risk enough to induce clinically relevant bias in intervention effect estimate; for continuous outcome data, plausible effect size (difference in means or standardized difference in means) among missing outcomes enough to induce clinically relevant bias in observed effect size; ‘As-treated’ analysis done with substantial departure of the intervention received from that assigned at randomization; potentially inappropriate application of simple imputation. Unclear risk is rated if insufficient reporting of attrition/exclusions to permit judgement of ‘Low risk’ or ‘High risk’ (e.g. number randomized not stated, no reasons for missing data provided); the study did not address this outcome

^7^ Reporting bias due to selective outcome reporting. Low risk is rated if the study protocol is available and all of the study’s pre-specified (primary and secondary) outcomes that are of interest in the review have been reported in the pre-specified way; the study protocol is not available but it is clear that the published reports include all expected outcomes, including those that were pre-specified (convincing text of this nature may be uncommon). High risk is rated if not all of the study’s pre-specified primary outcomes have been reported; one or more primary outcomes is reported using measurements, analysis methods or subsets of the data (e.g. subscales) that were not pre-specified; one or more reported primary outcomes were not pre-specified (unless clear justification for their reporting is provided, such as an unexpected adverse effect); one or more outcomes of interest in the review are reported incompletely so that they cannot be entered in a meta-analysis; the study report fails to include results for a key outcome that would be expected to have been reported for such a study. Unclear risk is rated if insufficient information to permit judgement of ‘Low risk’ or ‘High risk’. It is likely that the majority of studies will fall into this category.

^8^ Bias due to problems not covered elsewhere in the previous points. Low risk is rated if the study appears to be free of other sources of bias. High risk is rated if had a potential source of bias related to the specific study design used; or has been claimed to have been fraudulent; or had some other problem. Unclear risk is rated if insufficient information to assess whether an important risk of bias exists; or insufficient rationale or evidence that an identified problem will introduce bias
